# Evaluation of the Fracture Load of Multilayer Zirconia Onlay Restorations with Different Yttria Contents After Thermomechanical Aging: An In Vitro Study

**DOI:** 10.3390/ma19163381

**Published:** 2026-08-08

**Authors:** Ayşe Rençber Kızılkaya, Kübra Bilge, Aybüke Kara

**Affiliations:** 1 Department of Prosthodontics, Faculty of Dentistry, Firat University, Elazig 23119, Turkey; ayserencber23@hotmail.com; 2 Department of Restorative Dentistry, Faculty of Dentistry, Firat University, Elazig 23119, Turkey; kubratnyl@gmail.com

**Keywords:** CAD/CAM, digital dentistry, fracture load, multilayer zirconia, onlay restoration, thermomechanical aging, Weibull analysis

## Abstract

This in vitro study compared the fracture load of multilayer zirconia onlay restorations with different yttria contents after thermomechanical aging. Standardized CAD/CAM-fabricated onlay restorations were produced from three multilayer zirconia materials: one color-gradient material (KATANA Zirconia UTML; 5Y-TZP) and two strength-gradient materials (KATANA Zirconia YML, 3–5Y-TZP; IPS e.max ZirCAD MT Multi, 4–5Y-TZP) (*n* = 10/group). The restorations were cemented onto three-dimensionally printed resin dies and subjected to 240,000 loading cycles and 5000 thermal cycles. Fracture load was measured using a universal testing machine, and the data were analyzed by one-way analysis of variance, Tukey’s post hoc test, and Weibull analysis (α = 0.05). Fracture load differed significantly among the materials (*p* = 0.017). The UTML group (395.73 N) exhibited a significantly lower fracture load than the YML group (618.19 N), whereas the ZirCAD MT Multi group (565.24 N) did not differ significantly from either of the other two groups. Weibull analysis revealed no significant differences in modulus among the groups, whereas the characteristic load of the UTML group was significantly lower than that of the other two materials (*p* = 0.002). Within the conditions of this in vitro study, the strength-gradient materials, which contain lower yttria concentrations in their underlying layers, showed higher fracture loads after thermomechanical aging than the color-gradient material. These findings are limited to the tested laboratory conditions and require clinical confirmation.

## 1. Introduction

The widespread adoption of digital scanning and the continued development of digital design and production systems have increased the use of zirconia in prosthetic dentistry [1,2]. Zirconia is a biomaterial that combines high strength with structural stability. The addition of 3 mol% yttria stabilizes the tetragonal phase at room temperature, but conventional 3Y-TZP remains limited in translucency [3].

Materials with a higher yttria content (4Y-TZP and 5Y-TZP) were therefore developed to improve translucency, although their fracture resistance is lower than that of 3Y-TZP. Multilayer zirconia materials were subsequently introduced to achieve a balance between esthetic and mechanical properties. These are produced in two configurations: color-gradient materials, in which only the shade varies between layers, and strength-gradient materials, in which the yttria content also differs across layers (3–5Y-TZP and 4–5Y-TZP) [4,5,6,7].

The advancement of intraoral scanners, CAD/CAM systems, and 3D printing technologies has increased the use of indirect restorative procedures and facilitated the fabrication of precise and reliable restorations [8]. As the clinical use of multilayer zirconia grows, its mechanical performance becomes correspondingly more important. Information on the fracture behavior of multilayer zirconia onlay restorations after thermomechanical aging nevertheless remains limited.

Dental materials are frequently evaluated under static laboratory conditions, which do not reproduce the complexity of the oral environment. Cyclic loading, thermal fluctuation, moisture and parafunctional habits all affect the long-term performance of restorative materials, and thermomechanical aging protocols are therefore used to approximate clinical service conditions and to provide a more realistic assessment of durability [9,10,11].

Weibull analysis provides clinically relevant information regarding the reliability of restorative materials in addition to conventional fracture load measurements. The Weibull modulus (m) describes the scatter of strength values between specimens, with higher values indicating greater reliability and structural consistency [12,13].

Because variations in dentin structure may influence fracture behavior, three-dimensionally printed resin dies have been proposed to improve standardization and reproducibility in laboratory studies [14].

The aim of this in vitro study was to compare the fracture load of onlay restorations fabricated from color-gradient and strength-gradient multilayer zirconia materials following thermomechanical aging.

The null hypothesis was that no significant difference would be observed in the fracture load of onlay restorations fabricated from color-gradient and strength-gradient multilayer zirconia materials after thermomechanical aging.

## 2. Materials and Methods

A sensitivity power analysis was performed using G*Power version 3.1 (Heinrich Heine University, Düsseldorf, Germany). Using the F-test family, fixed-effects one-way ANOVA, three groups, a total sample size of 30 specimens, an alpha level of 0.05, and a statistical power of 85%, the minimum detectable effect size was calculated as Cohen’s f = 0.64. Therefore, the study was adequately powered to detect large between-group differences, whereas smaller effects may not have been detected.

This study evaluated the fracture load of onlay restorations fabricated from three multilayer zirconia materials with different yttria contents following thermomechanical aging: one color-gradient material (KATANA Zirconia UTML, 5Y-TZP; Kuraray Noritake) and two strength-gradient materials (KATANA Zirconia YML, 3–5Y-TZP; Kuraray Noritake and IPS e.max ZirCAD MT Multi, 4–5Y-TZP; Ivoclar). The materials are described in Table 1. These are representative multilayer zirconia systems with different yttria configurations, all indicated for monolithic restorations. Monolithic 3Y-TZP was not included because its limited translucency restricts its use in esthetic partial-coverage restorations such as onlays [3], and its composition is already represented within the gradient structure of the YML material. The three systems were selected as commercially available representatives of each configuration.

A master model was obtained by preparing an onlay cavity on an artificial maxillary right first premolar (FDI #14) using a chamfer diamond bur (Frasaco GmbH, Tettnang, Germany) [15]. The onlay preparation consisted of a 2.5 mm isthmus width, a 2 mm pulpal depth, and mesial and distal proximal boxes with a width of 1.5 mm, including the buccal cusp. A surveyor (Dental Surveyor; Unident, Istanbul, Turkey) was used to identify potential undercuts. The prepared model was digitized using a laboratory (extraoral) scanner (inEos X5; Dentsply Sirona, Charlotte, NC, USA) with inLab CAD SW 20.0.4 (Dentsply Sirona, 2021). The scan data were exported as a Standard Tessellation Language (STL) file.

**Figure 1:**
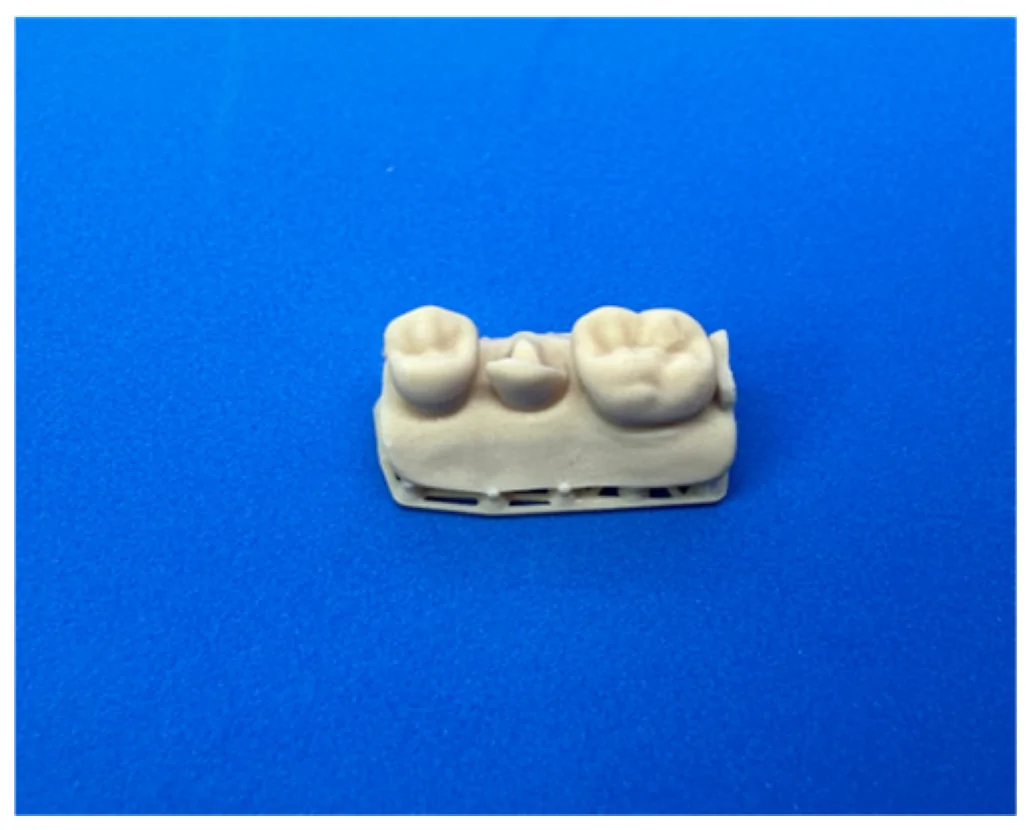
3D Printed Model of Onlay Cavity Preparation.

Die replicas were fabricated from this file with a Digital Light Processing (DLP) 3D printer (Freeform Pro 2; ASIGA, Anaheim Hills, Anaheim, CA, USA) (Figure 1). A resin with an elastic modulus of 2.5 GPa was used, and post-polymerization was carried out in an Otoflash device under a nitrogen atmosphere. Thirty resin dies were fabricated (*n* = 10 per group). Onlay restorations were designed using the same software (inLab CAD SW 20.0.4; Dentsply Sirona), and the cement space was set at 30 µm [16]. This value corresponds to the ideal cement film thickness reported for CAD/CAM restorations and was applied identically to all groups. To standardize the yttria distribution between restorations, all specimens were positioned in the same orientation and aligned with the same region of their respective blanks during the CAD design process. KATANA Zirconia UTML and KATANA Zirconia YML restorations were milled from 18 mm (T18) multilayer zirconia disks, whereas IPS e.max ZirCAD MT Multi restorations were milled from 16 mm multilayer zirconia disks. During the CAD/CAM nesting procedure, the occlusal aspect of each onlay coincided with the incisal (enamel) layer of the multilayer zirconia disk. The restoration body extended progressively into the underlying transition and body (or dentin) layers according to each manufacturer’s multilayer configuration. The restorations were milled with a 5-axis milling unit (inLab MC X5; Dentsply Sirona) in standard mode, and the burs were replaced after each group to limit the effect of bur wear. All restorations were sintered in a sintering furnace (inLab Profire; Dentsply Sirona) according to the manufacturers’ instructions.

**Figure 2:**
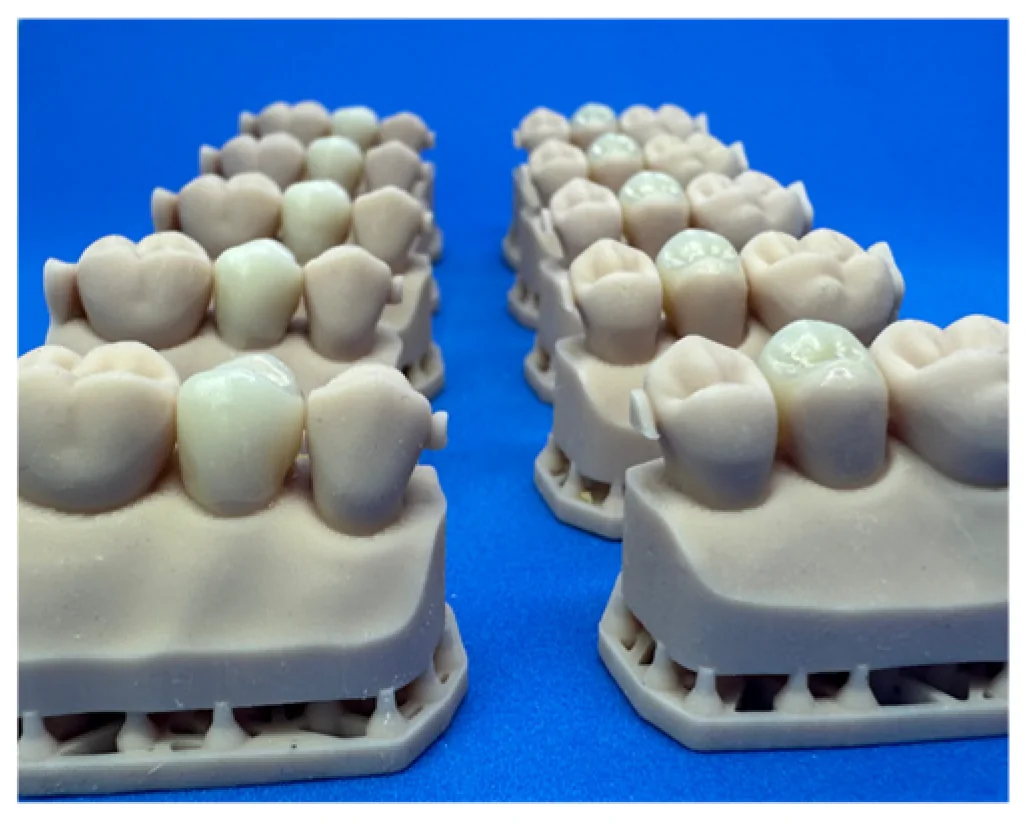
Cemented Onlay Restoration Group.

The die surfaces were airborne-particle abraded with 50 µm aluminum oxide and then cleaned in an ultrasonic water bath. As the printed resin dies contain no mineral phase, retention was obtained micromechanically through airborne-particle abrasion; all subsequent cementation steps followed the manufacturer’s instructions without modification. The intaglio surfaces of the restorations were abraded under the same conditions, in accordance with the manufacturer’s instructions. A dual-cure resin cement (Panavia SA Cement Universal; Kuraray Noritake) was then applied to each intaglio surface. The restorations were seated on the corresponding dies under a constant load of 25 N using a universal testing machine (MTS Criterion Model 42; MTS Systems, Eden Prairie, MN, USA). Excess cement was removed, and the restorations were light-polymerized using an LED curing unit (LED.B, Guilin Woodpecker Medical Instrument Co., Guilin, China) with an output intensity of 1470 mW/cm^2^ for 20 s (Figure 2). Immediately afterwards, an oxygen-inhibiting gel (Oxyguard II; Kuraray Medical Co., Tokyo, Japan) was applied for 4 min to protect the marginal cement from oxygen during the subsequent self-curing phase of the dual-cure cement, in accordance with the manufacturer’s instructions. The specimens were then stored at 37 °C and 100% humidity for 24 h before aging.

The cemented restorations were embedded in self-curing acrylic resin (SC Self Cure; Imicryl, Konya, Turkey) within a mold that served as the specimen holder for the chewing simulator. A dual-axis chewing simulator (Esetron Smart Robotechnologies, Mod Dental, Ankara, Turkey) subjected the specimens to 240,000 loading cycles, corresponding to approximately one year of clinical service [17], under a load of 50 N and a frequency of 1.7 Hz. Thermal aging was performed in a thermal cycler (Julabo, Seelbach, Germany), in which the specimens were subjected to 5000 thermal cycles between 5 °C and 55 °C in distilled water, with a dwell time of 20 s at each temperature and a transfer time of 10 s between the two baths. A 6 mm-diameter metal sphere was used to simulate the opposing tooth [17]. The vertical and horizontal movements of the sphere were set at 2 mm and 1 mm, respectively. All 30 specimens survived thermomechanical aging intact, with no cracking, chipping or debonding observed, and all were included in the fracture test.

**Figure 3:**
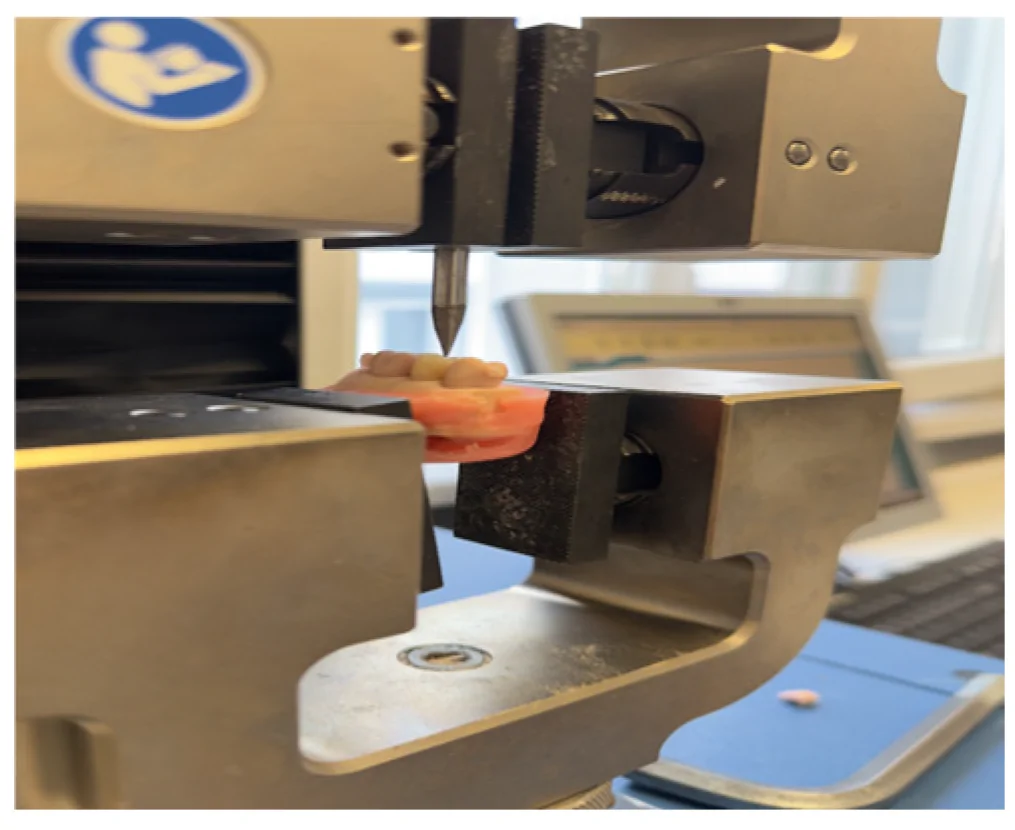
Specimen Placed in Testing Machine (Instron).

Following thermomechanical aging, fracture testing was performed using a universal testing machine (Instron, Canton, MA, USA) at a crosshead speed of 1 mm/min. Each specimen was mounted with its long axis parallel to the load axis, and the load was applied to the central fossa of the occlusal surface using a stainless-steel indenter with a conical tip 1.5 mm in diameter, without an interposed medium. Compressive load was applied to the occlusal surface until fracture occurred, and the maximum fracture load (N) was recorded (Figure 3).

Statistical analyses were performed using SPSS Version 22 (IBM Corp., Armonk, NY, USA). The normality of data distribution was assessed using the Shapiro–Wilk test and the homogeneity of variances using Levene’s test. One specimen in the YML group was identified as a statistical outlier and excluded, so that 29 specimens were included in the statistical evaluation. Since the data met the assumptions of normality and homogeneity of variance, one-way analysis of variance (ANOVA) was used to compare fracture load values among the groups, followed by Tukey’s post hoc test for pairwise comparisons. The significance level was set at *p* < 0.05.

The variability of fracture load values among specimens was further evaluated using Weibull analysis performed with Minitab 17 (Minitab Inc., State College, PA, USA). Weibull parameters were estimated using the maximum likelihood method. The shape (m) and scale (σ_0_) parameters were compared using the chi-square test. Statistical significance was set at *p* < 0.05.

## 3. Results

After thermomechanical aging, fracture load differed significantly between the onlay material groups (*p* = 0.017). The results are summarized in Table 2.

The fracture load of the UTML group was significantly lower than that of the YML group (*p* = 0.020). The ZirCAD MT Multi group did not differ significantly from either the UTML group (*p* = 0.057) or the YML group (*p* = 0.726).

The lowest individual value was recorded in the UTML group (216.15 N) and the highest in the ZirCAD MT Multi group (712.04 N). The mean values, in ascending order, were as follows: UTML (395.73 N), ZirCAD MT Multi (565.24 N), and YML (618.19 N) (Table 2).

**Figure 4:**
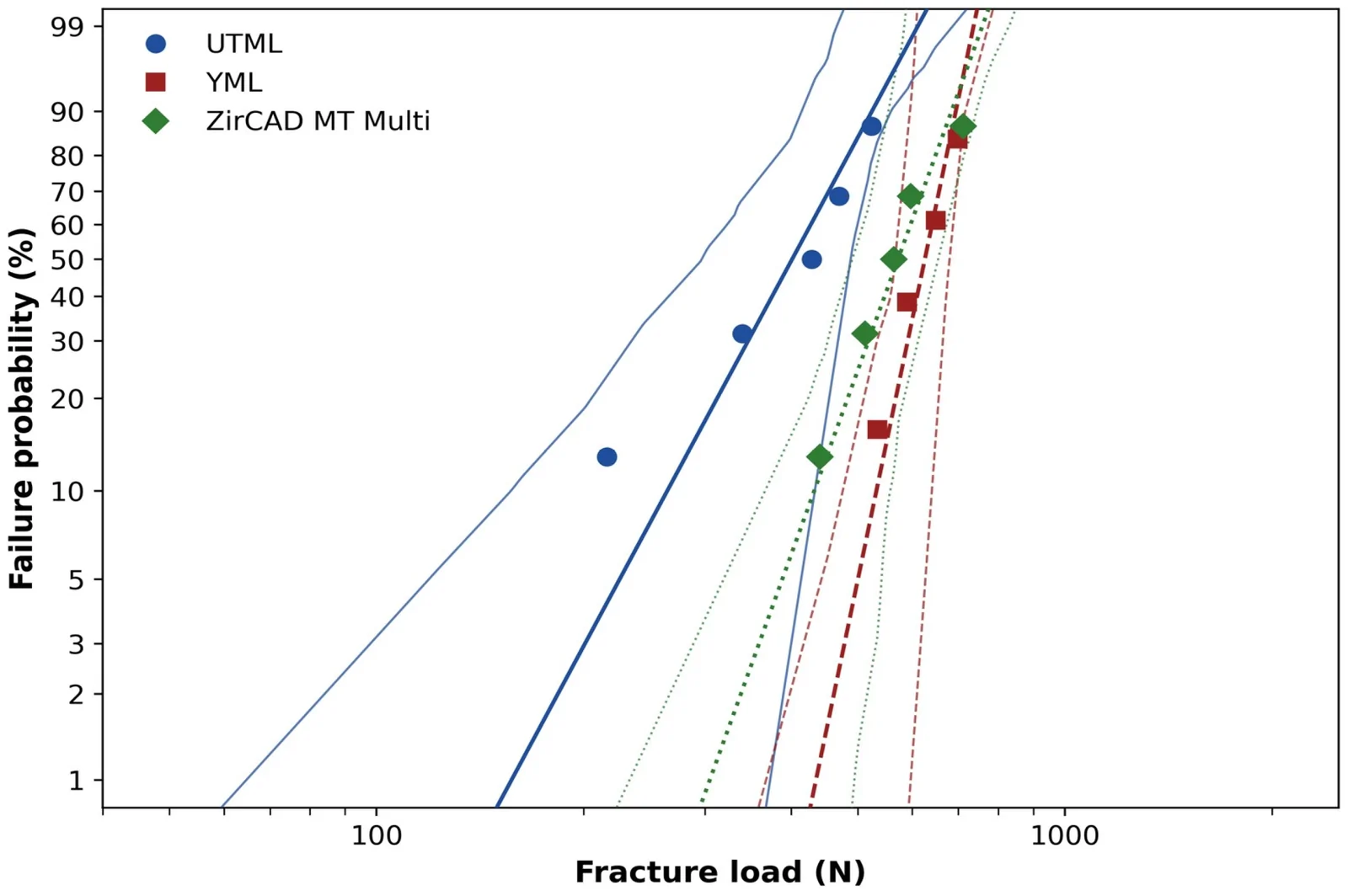
Probability Plot of Fracture Values.

The Weibull modulus reflects the scatter of fracture loads within a group: a high modulus indicates closely grouped values and therefore consistent behavior, whereas a low modulus indicates wide scatter. YML material has the highest Weibull modulus value of 11.620 and UTML material has the lowest Weibull modulus value of 4.508 (Figure 4). The corresponding 95% confidence intervals are given in Table 3. The modulus values did not differ significantly between the materials (*p* = 0.218).

The scale parameter (σ_0_), or characteristic load, is the load at which 63.2% of specimens are expected to fail and is closely related to the mean fracture load. A higher scale value therefore indicates a greater characteristic load, whereas variability is described by the modulus. The scale values differed significantly among the materials (*p* = 0.002), with significant differences observed between the UTML group and the other material groups. The results are summarized in Table 3.

## 4. Discussion

The present study evaluated the fracture load of multilayer zirconia onlay restorations with different yttria contents following thermomechanical aging. Significant differences in fracture load were observed among the tested materials. Therefore, the null hypothesis was rejected. The UTML group exhibited a significantly lower fracture load than the YML group, whereas the ZirCAD MT Multi group occupied an intermediate position and did not differ significantly from either material. No significant difference was found between the YML and ZirCAD MT Multi groups.

These differences can be explained by the yttria-dependent phase composition of zirconia. In 3Y-TZP, the predominantly tetragonal microstructure undergoes a stress-induced tetragonal-to-monoclinic transformation at the crack tip, and the associated volume expansion opposes crack propagation through transformation toughening. As the yttria content rises to 4 mol% and 5 mol%, the proportion of the stable cubic phase increases; translucency improves, but the capacity for transformation toughening falls and fracture resistance decreases accordingly [3]. Among the multilayer materials evaluated in the present study, the fully 5Y-TZP UTML is a color-gradient material that is multilayered only in shade, whereas the strength-gradient YML (3–5Y-TZP) and ZirCAD MT Multi (4–5Y-TZP) incorporate lower-yttria, higher-strength compositions in their cervical and dentin regions. These tougher underlying layers are consistent with the higher fracture loads recorded for the two strength-gradient groups, and the integrity of the interfaces between adjacent layers is likely to contribute as well [18].

Pöppel et al. compared the fracture load of four three-unit fixed dental prostheses made from multilayer zirconia with varying yttria contents (KATANA HTML, UTML, STML, YML) after 5 years of aging, and observed the maximum fracture load value (716.9 N) in HTML (3Y-TZP), followed by YML (3–5Y-TZP) at 701.8 N. The highest mean value was found in the HTML group (629.6 N), followed by the YML group (623.6 N) [19]. In the present study, after 1 year of aging, the highest mean fracture load value (618.19 N) among the onlay restorations was observed in the 3–5Y-TZP (YML) group. These findings suggest that multilayer zirconia materials containing lower yttria content in at least one layer may provide adequate mechanical performance after aging regardless of differences in restoration design.

In a study by Winter et al., the mechanical properties of different layers within multilayer zirconia materials (IPS e.max ZirCAD Prime, Optimill Multilayer 3D, Ceramill Zolid FX multilayer) were evaluated following thermal aging. The authors reported that the mechanical properties varied significantly according to the position of the layer within the zirconia blank, emphasizing that the location of the restoration within the multilayer block should be considered because of its influence on mechanical performance [20]. In the present study, however, no significant difference in fracture load was observed between the 3–5Y-TZP (YML) and 4–5Y-TZP (ZirCAD MT Multi) onlay restorations. Despite their different layer architectures, the two materials therefore performed comparably under the present loading conditions. However, as these materials are produced by different manufacturers, this finding cannot be attributed to yttria configuration alone. Nevertheless, further studies evaluating the fracture behavior of individual layers and different restoration positions within multilayer zirconia blanks are needed to better clarify the influence of layer composition on fracture resistance.

Kaizer et al. investigated the interfacial strength of newer multilayer zirconia materials, including UTML-5Y-PSZ, STML-4Y-PSZ, and ML-3Y-PSZ. They observed that an increase in yttria content led to a decrease in fracture load. The failure stress of multilayer beams was consistently lower than their enamel or dentin counterparts, regardless of the material tested. This difference suggests that the interfaces between chromatic layers may be weaker [18]. These findings are consistent with the results of the present study, in which the fully 5Y-TZP (UTML) material exhibited the lowest fracture load among the tested groups, suggesting that both increased yttria content and the integrity of the interfaces between adjacent layers may influence the mechanical performance of multilayer zirconia restorations.

Strasser et al. investigated the microstructure and mechanical properties of various multilayer zirconia blanks and reported significant differences primarily in the intermediate layers of the tested materials. The authors emphasized that, in addition to restoration dimensions, the milling position within multilayer zirconia blanks should be carefully considered because of its potential influence on mechanical performance [21]. These findings further emphasize that, in addition to yttria content, the position of the restoration within a multilayer zirconia blank may influence its mechanical behavior. In the present study, all restorations were nested in an identical position within the blank; the influence of milling position was therefore not evaluated and remains to be investigated.

Badr et al. compared the fracture resistance of monolithic and multi-yttria-layered zirconia crowns with different yttria contents following thermocycling and thermo-mechanical loading and reported that increasing the yttria content significantly reduced fracture resistance. They also showed that a higher yttria concentration in the occlusal portion reduced fracture resistance, and that stronger lower-yttria layers in the cervical region did not fully compensate for this reduction [22]. These findings are consistent with the results of the present study. Similarly, the fully 5Y-TZP UTML group exhibited a significantly lower fracture load than the YML (3–5Y-TZP) group, which contains lower yttria concentrations in its underlying layers, and the lowest mean fracture load overall. In the present study the occlusal aspect of each restoration coincided with the 5Y-TZP incisal layer in all three materials, whereas the underlying layers differed; the higher fracture loads of the YML and ZirCAD MT Multi groups are therefore consistent with a contribution from their lower-yttria underlying layers. As fractographic analysis was not performed, the region governing failure could not be identified.

A recent narrative review similarly concluded that a higher yttria content in the incisal region enhances translucency at the expense of mechanical performance, whereas lower yttria concentrations in the cervical and dentin regions improve it [23]. This is consistent with the present findings, in which the YML (3–5Y-TZP) and ZirCAD MT Multi (4–5Y-TZP) groups, both containing lower yttria concentrations in their underlying layers, demonstrated higher fracture loads than the fully 5Y-TZP UTML group.

In a study evaluating the mechanical performance of ultra-thin occlusal veneers fabricated from zirconia ceramics with different translucencies, 3Y-TZP demonstrated significantly higher fracture resistance than 5Y-TZP after thermo-mechanical aging, while 4Y-TZP and multilayer zirconia exhibited satisfactory mechanical performance. The authors also reported that all zirconia materials maintained clinically acceptable mechanical properties after artificial aging and that fracture resistance tended to decrease with increasing yttria content [24]. These findings are consistent with those of the present study, in which the fully 5Y-TZP UTML group exhibited the lowest mean fracture load and differed significantly from the YML (3–5Y-TZP) group. Taken together, these results indicate that yttria content remains a primary determinant of mechanical performance across different restoration designs, with lower-yttria phases contributing substantially to fracture resistance.

The fracture loads reported here should be interpreted with caution clinically, since masticatory forces vary considerably between individuals and are higher in the presence of parafunctional habits such as bruxism. Furthermore, although 240,000 loading cycles are commonly used in laboratory simulations to approximate clinical service, the actual clinical service time may vary depending on individual chewing frequency, dietary habits, and parafunctional activity. Therefore, the present findings should be interpreted within the limitations of an in vitro study.

This study has several limitations. The restorations were cemented onto three-dimensionally printed resin dies rather than natural teeth; these dies were chosen to standardize specimen geometry and to avoid the anatomical variability of natural teeth, which improves reproducibility. Their elastic modulus (approximately 2.5 GPa) is nevertheless much lower than that of dentin (approximately 15–20 GPa), which may have affected stress distribution and the absolute fracture loads. Moreover, adhesion to the printed resin dies was obtained micromechanically, whereas bonding to enamel and dentin additionally involves chemical interaction; the absolute fracture loads reported here may therefore be lower than those obtainable on natural teeth. Minor variation arising from the manufacturing process cannot be excluded either. Because identical dies were used for all groups, however, the comparison between materials was not affected. In addition, the materials were obtained from two manufacturers; factors other than yttria content—including grain size, additives, layer architecture, sintering conditions, surface defects and residual stresses—may therefore also have influenced the results, and the observed differences cannot be attributed to yttria content alone. As no non-aged control group was included, the effect of aging itself could not be evaluated, and the comparison is limited to the materials under identical aged conditions. Fracture load was also assessed with a single-load-to-failure test, so the results should be interpreted within the constraints of an in vitro design. Future studies on natural tooth substrates, comparing systems from a single manufacturer and examining fracture patterns and long-term fatigue behavior, would help to confirm these findings.

## 5. Conclusions

After thermomechanical aging, fracture load differed significantly between the multilayer zirconia onlay restorations tested. The 5Y-TZP material (UTML) withstood a significantly lower load than the 3–5Y-TZP material (YML), whereas the 4–5Y-TZP (ZirCAD MT Multi) material did not differ significantly from either group. Weibull analysis gave the highest modulus for YML and the lowest for UTML, but these differences were not significant. Within the limitations of this in vitro study, the strength-gradient materials, which contain lower yttria content in their underlying layers, withstood higher loads than the fully 5Y-TZP material. Because these results were obtained under standardized laboratory conditions on resin dies, they cannot be extrapolated to clinical performance without further clinical investigation.

## Figures and Tables

**Table 1:** Technical profiles of materials used in this study.

Material	Zirconia Type	Composition	Manufacturer
KATANA Zirconia UTML	5Y-TZP (color-gradient multilayer)	ZrO_2_ + HfO_2_ (87–92%), Y_2_O_3_ (8–11%), Other oxides (0–2%)	Kuraray Noritake Dental Inc., Tokyo, Japan
KATANA Zirconia YML	3–5Y-TZP (strength-gradient multilayer)	Multilayer zirconia composed of 5Y-TZP and 3Y-TZP layers with a gradient yttria distribution	Kuraray Noritake Dental Inc., Tokyo, Japan
IPS e.max ZirCAD MT Multi	4–5Y-TZP (strength-gradient multilayer)	ZrO_2_ (86.0–93.5%), Y_2_O_3_ (6.5–8.0%), HfO_2_ (≤5.0%), Al_2_O_3_ (≤1.0%), Other oxides ≤ 1.0%	Ivoclar, Schaan, Liechtenstein

Y_2_O_3_: Yttrium oxide, HfO_2_: Hafnium oxide, ZrO_2_: Zirconium oxide, Al_2_O_3_: Aluminum oxide, 3Y-TZP: 3 mol% yttria-stabilized tetragonal zirconia polycrystal, 4Y-TZP: 4 mol% yttria-stabilized tetragonal zirconia polycrystal, 5Y-TZP: 5 mol% yttria-stabilized tetragonal zirconia polycrystal.

**Table 2:** Fracture load of the material groups.

Material	Fracture Load (N), Mean ± SD
**UTML**	395.73 ± 120.82 ^a^
**YML**	618.19 ± 71.47 ^b^
**ZirCAD MT Multi**	565.24 ± 101.11 ^ab^
*p* value	0.017 *

Different superscript letters indicate statistically significant differences between groups (*p* < 0.05). * Statistically significant at *p* < 0.05.

**Table 3:** Weibull parameters of the tested zirconia materials.

Material	Shape (m)		Scale (N)	
	Shape (%95 CI)	SD	Scale (%95 CI)	SD
**UTML**	4.508 (2.14–9.493)	1.713	435.729 (355.486–534.085) ^a^	45.249
**YML**	11.620 (5.410–24.956)	4.532	646.220 (591.305–706.234) ^b^	29.280
**ZirCAD MT Multi**	6.719 (3.451–13.084)	2.285	604.537 (526.455–694.199) ^b^	42.656
χ^2^ statistic	3.044		12.102	
*p* value	0.218		0.002	

Chi-square test. Values with the same superscript letter are not significantly different (*p* > 0.05). SD: standard deviation.

## Data Availability

The original contributions presented in this study are included in the article. Further inquiries can be directed to the corresponding author.

## Abbreviations

The following abbreviations are used in this manuscript:

3-YTZP	3 mol% yttria-stabilized tetragonal zirconia polycrystal
4-YTZP	4 mol% yttria-stabilized tetragonal zirconia polycrystal
5-YTZP	5 mol% yttria-stabilized tetragonal zirconia polycrystal
CAD/CAM	Computer-Aided Design/Computer-Aided Manufacturing
DLP	Digital Light Processing
STL	Standard Tessellation Language
ANOVA	Analysis of Variance
SD	Standard Deviation
CI	Confidence Interval
LED	Light-Emitting Diode
UTML	Ultra Translucent Multi Layer
YML	Yttria Multi Layer
